# Palm Foliage as Pathways of Pathogenic *Botryosphaeriaceae* Fungi and Host of New *Lasiodiplodia* Species from Mexico

**DOI:** 10.3390/pathogens10101297

**Published:** 2021-10-08

**Authors:** Clovis Douanla-Meli, Andreas Scharnhorst

**Affiliations:** 1 Julius Kühn Institute (JKI)—Federal Research Centre for Cultivated Plants, Institute for National and International Plant Health, Messeweg 11-12, 38104 Braunschweig, Germany; 2 Regierungspräsidium Gießen—Dezernat 51.4, Pflanzenschutzdienst Hessen, Schanzenfeldstr. 8, 35578 Wetzlar, Germany; andreas.scharnhorst@rpgi.hessen.de

**Keywords:** global trade, cut greenery, invasive fungal species, new diseases

## Abstract

Tropical palm foliage is increasingly imported to satisfy the steady growing demand in European floristry. This palm foliage presumably carries along diverse fungi whose taxonomic and functional diversity have not been addressed so far. The present study investigated *Botryosphaeriaceae* fungi associated with the foliage of palm species *Chamaedorea elegans*, *C. metallica*, *C. seifrizii*, *Dypsis lutescens* and *Lodoicea maldivica* imported from Mexico. Five species were identified using combined morphological characterisation and multilocus phylogenetic analyses based on *ITS*, *TEF-1α*, *TUB2* and *RPB2*. In addition to *Endomelanconiopsis endophytica*, *Lasiodiplodia brasiliensis* and *L. euphorbicola*, two new species, namely, *L. lodoiceae* sp. nov. and *L. mexicanensis* sp. nov, are proposed. Apart from *E. endophytica*, mostly known as endophyte, *L. brasiliensis* and *L. euphorbicola* are responsible for different rot diseases and the dieback of important tropical crop plants. In pathogenicity tests on the temperate pome fruits apple *(Malus domestica)* and pear (*Pyrus communis*), all six *Botryosphaeriaceae* species induced necrotic lesions at different degrees of severity, with highest the aggressiveness from *L. euphorbicola* and *L. mexicanensis* on apple and from *L. mexicanensis* on pear. The results indicate that tropical palm foliage can be a pathway of potentially pathogenic fungi that may give rise to concerns with regard to plant health in the destination countries.

## 1. Introduction

Tropical palms (Arecales, *Arecaceae*) naturally inhabiting warm climate regions [1] are currently distributed worldwide, mostly due to their use as ornamental plants and important accessory in interior decoration and in floristry. There is perpetual increasing demand of tropical palm foliage in Europe and thus the import from third countries [2]. Generally, the import should comply with existing phytosanitary requirements aiming at preventing the introduction and spread of harmful organisms in the EU through plant import (Regulation EU 2016/2031, 2016). If the introduction of the entire palm plants is regulated, the trade of palm foliage is going on freely. Furthermore, it has not yet been investigated which fungi are associated and which phytosanitary risk may be related to this foliage.

Tropical plants are recognized to support the highest-level diversity of endophytes and latent pathogenic fungi [3]. Numerous studies disclosed that different fungal assemblages are particularly associated with palms [4,5,6]. Hereupon, it can be ascertained that imported palms bring along considerable fungal diversity. Extensive studies on some tropical palms [4,7] found out that fungal communities potentially differ from that on temperate palms [8,9]. In this respect, tropical palms likely represent the pathway of numerous tropical fungal species exotic to Europe. Common pathogenic fungi associated to palm diseases mostly belong to the families *Pestalotiopsidaceae* and *Glomerellaceae* and have an immense impact on many trees’ fruit crops [10,11,12]. Other families, such as *Botryosphaeriaceae*, are also reported to be associated with palms [13,14,15].

*Botryosphaeriaceae* fungi (Ascomycetes, Dothideomycetes) are worldwide distributed as endophytes but mostly as pathogens affecting various crops and forest plants [16,17,18,19,20,21,22,23,24,25,26]. As endophytes, they asymptomatically colonise all plant tissues and can be found as saprotrophs on dead plant material. This endophytic phase may be indeed a latent phase from which many *Botryosphaeriaceae* species instantly become pathogenic, causing symptoms such as leaf lesions, fruit and root rot, dieback and cankers [27]. One of the widespread and most studied genera is *Lasiodiplodia*, comprising many plant-pathogenic species causing yield loss to diverse tropical and even temperate crops [20,22,24,25,26,28]. Two species of this genus, *L. theobromae* and *L. pseudotheobromae*, are also implicated in human opportunistic infections as keratitis, subcutaneous infections, sinusitis and pneumonia [29,30,31]. Several *Lasiodiplodia* species are ubiquitous and plurivorous, and the most geographically distributed *L. theobromae*, for example, affects more than 280 plant species [19,22,28,32,33]. Other species still have restricted host range and geographical distribution [23,26,27,28]. However, it is worth noting that global trade, resulting in the movement of infected plant material, is contributing to the rapid spread of many of these originally restricted-range fungal species [34,35,36]. In this respect, *Lasiodiplodia* currently mostly distributed in the tropics is expected to be moving into warm temperate regions. There is no investigation on the involvement of palm foliage in this phenomenon and its possible implication in the issue of exotic and new fungal plant diseases. 

Trade of cut foliage from third countries to Europe implies the movement of a large quantity of diverse plant material. To prevent the introduction of harmful organisms, it is important to identify the possible involved risk products. In this regard, we recently launched a study targeting the fungal communities associated with diverse cut foliage imported from third countries to Germany. It is hypothesized that this plant material, asymptomatic or symptomatic, can be pathways for the transition of endophytic and pathogenic fungi to susceptible hosts. During fungal isolation, palms showed high colonisation by *Botryosphaeriaceae* fungi, and the purpose of the present study was to characterize these isolates using morphology and phylogeny based on four molecular markers. A further objective was the preliminary evaluation of the risk associated to these species by testing their pathogenicity on two temperate pome fruits.

## 2. Results

### 2.1. Phylogenetic Analysis and Species Identification

**Figure 1:**
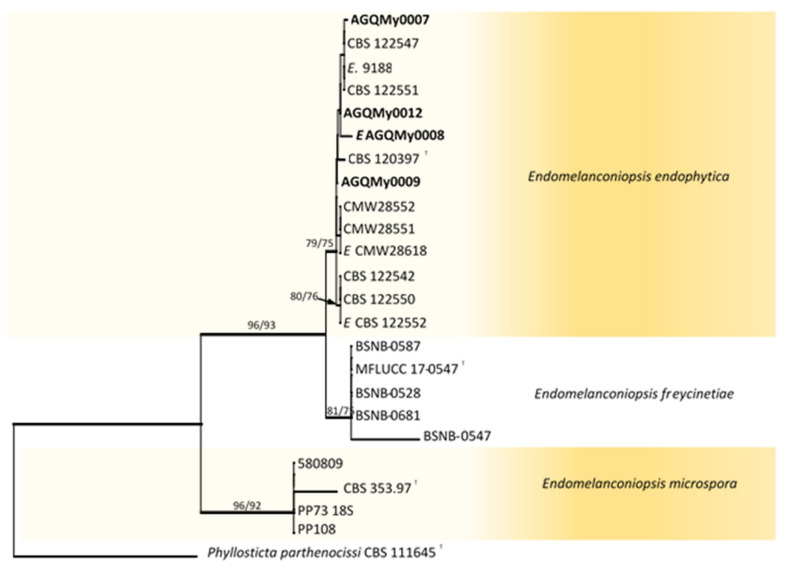
RAxML tree reconstructed by analysing the concatenated dataset of *ITS* and *TEF-1α* showing the phylogenetic affinities of isolates from palm foliage from Mexico within the genus *Endomelanconiopsis*. Bayesian posterior probabilities (BPP) greater than 0.95 are represented by thick lines. Maximum likelihood bootstrap values (MLB) higher than 60% (based on 1000 replicates) are displayed at the nodes (MLB/MPB). Colour shading indicate well-delimited phylogenetic species. The tree is rooted to *Phyllosticta parthenocissi* (CBS 111645). Isolates from palm foliage are in bold and ex-type isolates are marked with ^T^.

**Figure 2:**
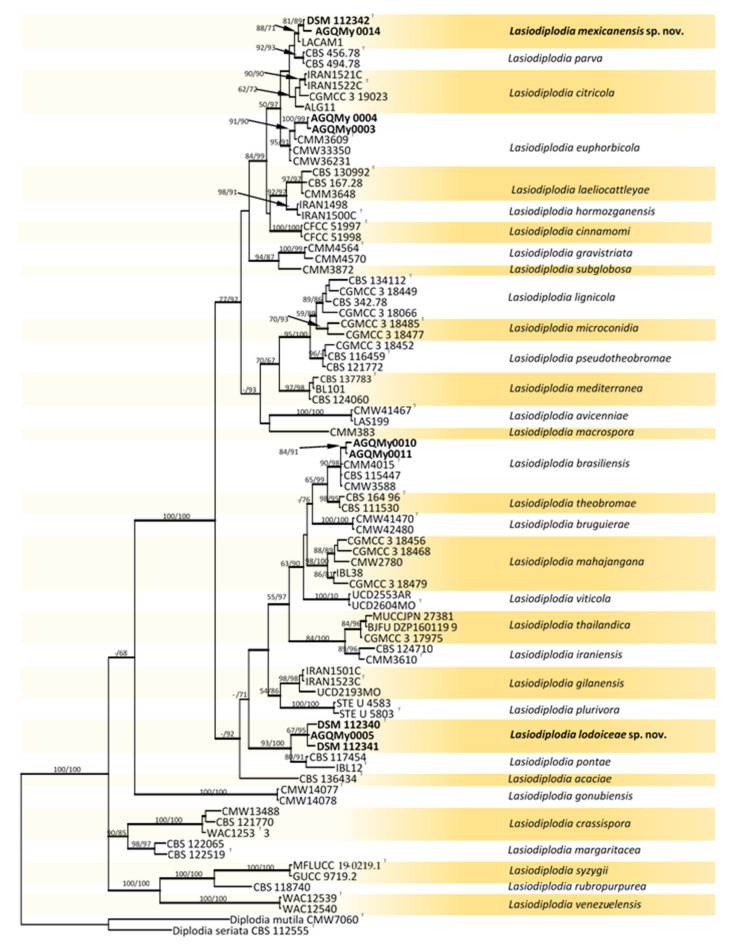
RAxML tree reconstructed by analysing the concatenated dataset of *ITS*, *TEF-1α*, *TUB2* and *RPB2* showing the phylogenetic affinities of isolates from palm foliage from Mexico within the genus *Lasiodiplodia*. Bayesian posterior probabilities (BPP) greater than 0.95 are represented by thick lines. Maximum likelihood bootstrap values (MLB) and maximum parsimony bootstrap (MPB) higher than 60% (based on 1000 replicates) are displayed at the nodes (MLB/MPB). Colour shading indicate well-delimited phylogenetic species. The tree is rooted to *Diplodia mutila* (CMW7060) and *Diplodia seriata* (CBS 112555). Isolates from palm foliage are in bold characters and ex-type isolates are marked with ^T^.

Fifty-eight *Botryosphaeriaceae* isolates obtained from palm foliage were grouped into seventeen morphospecies. The *TEF-1α* sequences of the representatives of morphospecies formed seven OTUs, out of which one identified as *Endomelanconiopsis* and six belonged to *Lasiodiplodia*. To identify OTUs at the species level, a concatenated *ITS* and *TEF-1α* dataset was analysed for *Endomelanconiopsis*. The alignment comprised 24 entries and *Phyllosticta parthenocissi* (CBS 111645) was added and used as an outgroup. From the 1043 characters, including gaps, 36 (3.5%) were parsimony informative. An MP analysis found 10,000 equally parsimonious trees of 248 steps with CI = 0.984, RI = 0949, RC = 0.933 and HI = 0.016). The Bayesian, ML and MP trees had identical topologies. Three highly supported clades were delimited corresponding to the currently known *Endomelanconiopsis* species and all isolates from palm foliage were grouped to *E. endophytica* (Figure 1). For the species identification of *Lasiodiplodia*, combined datasets of four loci, *ITS*, *TEF-1α*, *TUB2* and *RPB2*, consisted of 83 *Lasiodiplodia* isolates. *Diplodia seriata* (CBS 112555) and *D*. *mutila* (CBS 112553) were included and used as outgroup taxa. The alignment contained 1687 characters including gaps, out of which 400 (23.7%) were parsimony informative. An MP analysis resulted in 11,921 equally parsimonious trees of 1170 steps with CI = 0.576, RI = 0.850, RC = 0.490 and HI = 0.424. The best-fit model TrN+I+G was selected for the combined data and used in the Bayesian analysis. The Bayesian and ML trees had identical topologies, the same as that of the 50% majority rule consensus of the MP trees. The RAxML tree is shown in Figure 2. RAxML trees resulting from the analysis of individual loci are shown in Figures S1–S4. The multigene tree was consistent with the recently revised global phylogeny of *Lasiodiplodia* [37]. The nine representatives of *Lasiodiplodia* isolates from palm foliage grouped in four subclades were well supported by all analyses. Two isolates were resolved in a clade including the ex-type of *L. euphorbicola* (CMM3652), two isolates clustered with *L. brasiliensis* (ex-type CMM 4015) and two isolates grouped with *Lasiodiplodia* sp. LACAM1 forming a clade closely related to *L. parva*. The remaining three isolates formed a distinct lineage sister to the ex-type of *L. pontae* (CMM1277). This lineage and the clade of *Lasiodiplodia* sp. LACAM1 are proposed to represent new species. Phylogenetic trees from individual loci greatly varied in topology. Excepting some basal nodes that were strictly resolved and constant among trees, the upper nodes differed among loci. The *TEF-1α* phylogeny had a better resolution, displaying roughly the same groupings as resolved with the four loci phylogeny. The two new *Lasiodiplodia* lineages were both resolved, albeit the isolate LACAM1 did not clearly group to the second lineage. The *ITS* and *RPB2* phylogenies (Figures S1 and S4) further supported these lineages as representing new *Lasiodiplodia* species. Additionally, the support for new species was provided by base-pairs sequence comparison with the ex-type of the closely related species.

### 2.2. Pathogenicity of *Botryosphaeriaceae* Species on Apple and Pear

**Figure 3:**
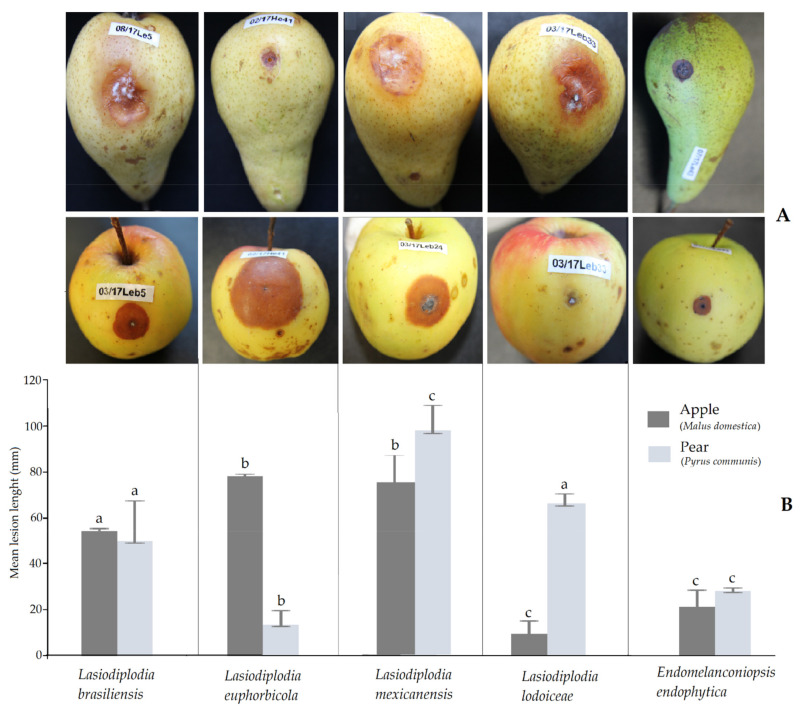
(**A**) Lesion caused by the six *Botryosphaeriaceae* species associated with palm foliage from Mexico on the two temperate pome fruits pear (*Pyrus communis*) (upper row) and apple (*Malus domestica*) (lower row). (**B**) Mean lesion length (mm). Bars above columns indicate standard errors of the mean and letters express significant differences. For each fruit, columns with the same letter do not differ significantly according to *t*-test at a = 0.05.

Pathogenicity tests were primarily conducted on fruits, but there is a prospect to carry on later further inoculation trials using seedlings. All isolates of the six *Botryosphaeriaceae* species tested on apple (*Malus domestica*) and pear (*P. communis*) fruits produced round necrotic lesions that progressed inside as rots. Sites on the fruit inoculated with sterile PDA as controls had restricted halos attributed to wound reactions. The pathogenicity was evaluated based on the size of the lesions developed from the inoculation point compared to the control. Confirmation was obtained with the fulfilment of Koch’s postulates by the successful re-isolation of the inoculated isolates. Isolates of same species produced different lesion lengths with a very wide difference among isolates of *L. mexicanensis* on apple. The severity of the isolates varied within fruit type (Figure 3). The highest aggressiveness recorded on apple was caused by *L. euphorbicola* followed by *L. mexicanensis*, which produced the largest necrotic lesions (mean up to 70 mm after 8 d). Moderate aggressiveness on apple was observed with *L. brasiliensis* and *E. endophytica*. On pear, the most aggressive species was *L. mexicanensis* followed by *L. lodoiceae* and *L. brasiliensis*. Moderate aggressiveness on pear was caused by *E. endophytica*. The lowest aggressiveness was caused by *L. lodoiceae* on apple and *L. euphorbicola* on pear. During infection on apple, mycelium of all species was mostly embedded in the necrotic area. Contrarily, three species *L. brasiliensis*, *L. mexicanensis* and *L. lodoiceae* externally produced a mycelial mass on pear.

### 2.3. Taxonomy

**Figure 4:**
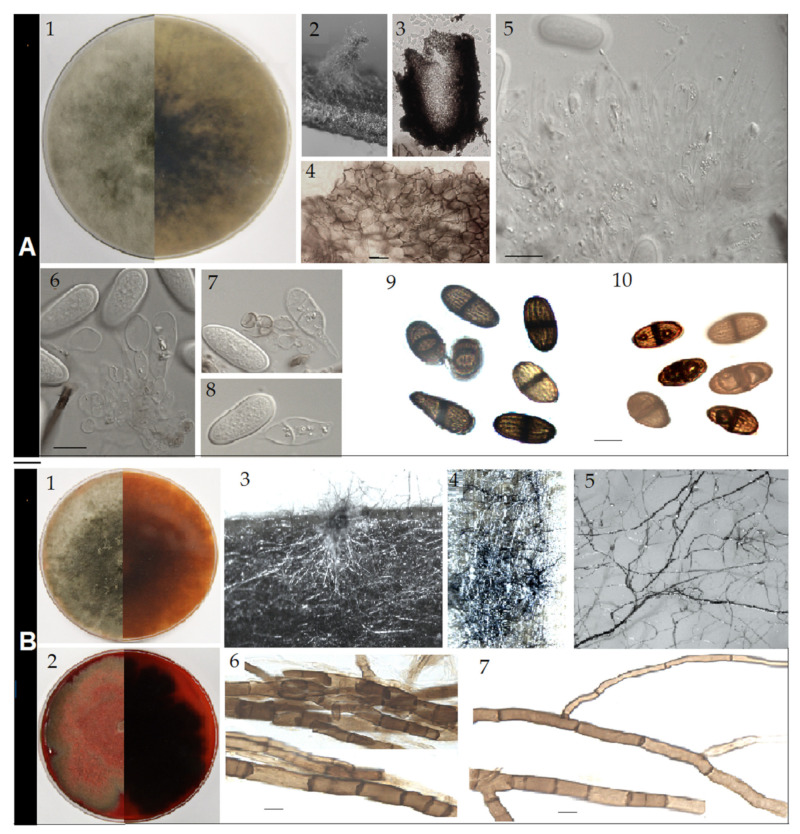
(**A**) *Lasiodiplodia lodoiceae* (Holotype DSM 112340). (**1**) Colony on PDA after 10 d at 25 °C showing above (left) and reverse (right) side; (**2**) Colony sporulating on WA pine needle; (**3**) Squash mount of conidiomata; (**4**) Cells-layer of conidiogenous wall; (**5**) Conidiogenous layer with developing conidia and paraphyses; (**6**) Conidiogenous cells; (**7**,**8**) Young, hyaline, thick-walled and often 1-septate conidia; (**9**,**10**). Mature, brown, 1-septate conidia with longitudinal striations, (**9**). from the type DSM 112340, (**10**). from isolate AGQMy0006. (**B**) *Lasiodiplodia mexicanensis* (Holotype DSM 112342). (**1**) Colony on PDA after 10 d at 25 °C. (**2**) Colony on PDA after 10 d at 35 °C. (**3**) Conidiomatal primordium. (**4**,**5**) Dark mycelial cords on pine needle and agar. (**6**,**7**) Hyphae from mycelial cords. Scale bars: 4 = 5 μm, 5, 6, 9 = 10 μm, scale bar of 6 applies to 7, 8 and of 9 applies to 10.

*Lasiodiplodia lodoiceae* C. Douanla-Meli, sp. nov. Figure 4A., MB 841286.

*Etymology:* the epithet refers to the host plant, the palm *Lodoicea maldivica*, from which the fungus was isolated. 

*Sexual form:* not observed. *Asexual form*: conidiomata up to 1.5 mm diameter and 2 mm height, pycnidial, stromatic, developing on pine needle SNA and WA after three weeks, covered with a scanty mycelial mat, mostly tubular, solitary or clustered (Figure 4A2), dark grey. Conidiomata also produced directly on agar medium, initially immerse and flat, and later becoming superficial, mucilaginous, upright, solitary, cylindrical with broadened base and attenuating toward the apex, dark and shiny. *Paraphyses* up to 60–75 × 2–3 µm (average 68.4 × 2.6 µm, n = 50), hyaline, smooth, filiforme and rounded at apex, unbranched emerging from and protruding out of the conidigeneous layer. *Conidiophores* absent. *Conidiogenous cells* up to 6–10 × 3–8 µm (average 8.6 × 5.1 µm, n = 50), holoblastic, hyaline, discrete, smooth, sugblobulose and thin-walled. *Conidia* initially hyaline, aseptate, but few prematurely one-septate, even when still attached to conidiogenous cells (Figure 4A7), with granula content, ellipsoid to ovoid, narrowing at one apex or both apexes broadly rounded; later at maturity, all one-septate with longitudinal striations, on one strain characteristically gutulate with one large gutule per cell (Figure 3A10), brown to dark brown, thick-walled, some noticeably tapered at one apex, 16.7–19.5 × 8.4–9.5 µm (average 18.1 × 8.9 µm, n = 50).

*Cultural characteristics*: colonies on PDA at 25 °C (Figure 4A1) early white, uniformly producing flattened dense mycelial mat, later greyish to brownish at the margin and grey greenish to dark grey in the centre, entire edge, margin flat, cottony, and centre slightly to strongly fluffy, grey to grey greenish. Growth reaching a maximal of 29.7 mm day^−1^. Colonies at 35 °C early sparse, flat, grey to dark grey, becoming grey-green at the centre, edge rough to irregular. Growth slower with maximum 22.3 mm day^−1^.

*Material examined.* Mexico, on leaves lesion of *Lodoicea maldivica* (*Arecaceae*), isolated 11 July 2017, Douanla-Meli (AGMy0002 Holotype, dried on pine needles; ex-type culture: DSM 112340. Additional material: Mexico, on leaves lesion of *Lodoicea maldivica*, isolated 20 July 2017, Douanla-Meli DSM 112341, AGQMy0005).

*Notes*: The four loci phylogeny resolved *L. lodoiceae* as a sister taxon of *L. pontae*. Both species can be, however, morphologically distinguished based on conidia characteristics. Conidia of *L. lodoiceae* showed septation at the immature stage and are at maturity smaller compared to the longer and larger conidia (16.4–26.4 × 9.6–15 µm) of *L. pontae*. Furthermore, the bi-gutulate character can distinguish the conidia of *L. lodoiceae* from those of *L. pontae*. The conidiogenous shape and size of *L. lodoiceae* differ from those of *L. pontae*. In addition, the production of reddish pigment at 35 °C characterizing *L. pontae* was not observed with *L. lodoiceae*. In base-pairs sequence comparison and nucleotide similarity (NS) between the ex-type culture of *L. pontae* (CMM1277) and the ex-type culture of *L. lodoiceae* (DSM 112340) based on alignable lenght, *L. lodoiceae* differs on *ITS* with one base pair (472/473; 99.8% NS) from *L. pontae* and both formed in ML tree (Figure S1), a group also including *L. gravistriata*, *L. subglobosa* and *L. macroscopora*. On *TEF-1α*, *L. lodoiceae* differs with 11 base pairs (265/276; 96% NS) and the ML analysis (Figure S2) placed it as a distinct lineage closely related to *L. pontae*. On *TUB2*, it differs from *L. pontae* with five base pairs and one indel (400/405; 98.8% NS) and the ML tree (Figure S3) placed both species as closely related. The *RPB2* sequence of *L. pontae* (CMM1277) was not available and the ML tree (Figure S4) placed *L. lodoiceae* as a distinct lineage with sister *L. plurivora*. 

*Lasiodiplodia mexicanensis* C. Douanla-Meli, sp. nov. Figure 4B, MB 841285. 

*Etymology*: the epithet refers to the country, Mexico, from where material of the host plant is originated.

*Sexual form*: not observed. *Asexual form*: no mature conidiomata developed on pine needle on PDA, MEA, Water Agar and SNA, even after six months under 12 h photoperiod and at various temperatures. The few conidiomatal primordia formed (Figure 4B3) did not further develop and mature. Meanwhile, under the 12 h photoperiod, thickened dark and frequently divided rhyzomorphic mycelium abundantly developed on agar and also invaded the pine needle (Figure 4B4,5). These mycelial cords were very brittle and composed of aggregated brown to dark brown hyphae. Individual hyphae were dichotomously branched, regularly septate, 4–9 µm diameter on the long trunk, with branches arising at acute angles and continuing as slender stalks of 2–3 µm diameter (Figure 4B6,7).

*Cultural characteristics*: colonies on PDA at 25 °C early white, producing uniformly flattened dense mycelial mat, later becoming greyish to brownish at the margin and grey greenish to dark grey in the centre, entire edge, margin flat and cottony and centre slightly to strongly fluffy, brownish to reddish pigment diffusing in agar (Figure 4A1). Growth faster, reaching 8 mm after 24 h, maximal growth rate of 30.4 mm day^−1^. Colonies at 35 °C early sparse, flat, grey to dark grey, becoming grey reddish to red after three days as the heavy production of pigment starts, characteristically zonate, edge rough to irregular. Pigment red to dark red, diffusing in agar and colouring the mycelium (Figure 4B2). Growth slower with maximum 23.5 mm day^−1^.

*Material examined.* Mexico, on leaves of *Chamaedorea seifrizii* (*Arecaceae*), isolated 7 June 2017, Douanla-Meli (AGQMy0015 Holotype, dried on pine needles; ex-type culture DSM 112342). Other isolate: Mexico, on leaves lesion of *C. seifrizii*, isolated 30 June 2017, Douanla-Meli AGQMy0014.

*Notes*: Four loci phylogenetic analyses grouped isolates of *L. mexicanensis* with the isolate LACAM1 on a well-supported clade (100% BPP, 88% MLB and 71% MPB). Closely related species to *L. mexicanensis* were *L. parva*, *L. citricola* and *L. euphorbicola,* respectively. In cultural characteristics, *L. mexicanensis* differs from all above related species by the production of reddish brown to red pigment and even at both 25 and 35 °C. In addition, the development in culture of rhizomorph-like structures characterizes *L. mexicanensis*. The conidial characters of isolates from *C. seifrizii* were not observed as they did not form mature conidiomata in culture. However, the description of the asexual form of the isolate LACAM1 is available in [25]. Phylogenetically, the relationship between *L. mexicanensis* and *L. parva* was poorly supported. *L. citricola* was resolved basal to the clade of *L. mexicanensis* and *L. parva* and *L. euphorbicola* was distantly resolved sister to the clade of *L. mexicanensis*, *L. parva* and *L. citricola*. In base-pairs sequence comparison and nucleotide similarity between the ex-type culture of *L. parva* (CBS 456.78) and ex-type culture of *L. mexicanensis* (DSM 112342), there were five base differences on *ITS* (525/530; 99.4% NS) and the ML analysis (Figure S1) placed it as a distinct lineage with 92% support (Figure S1). On *TEF-1α*, *L. mexicanensis* differs with three base pairs (301/304; 99% NS) from *P. parva* and in the ML tree (Figure S2) *L. mexicanensis* grouped to *P. parva* in an unresolved clade. On *TUB2* and *RPB2*, *L. mexicanensis* is identical to *L. parva*. The ML trees (Figures S3 and S4) did not resolved it from the closest species *L. parva*, *L. citricola* and *L. euphorbicola*. The ex-type culture of *L. mexicanensis* (DSM 112342) had similar nucleotide difference patterns with the ex-type cultures of *L. citricola* (IRAN1522C) and *L. euphorbicola* (CMM3609): 476/477 (99.8% NS) and 500/503 (99.4% NS) on *ITS*, respectively; 302/305 (99% NS) and 445/455 (97.8% NS) on *TEF-1α*, respectively; 422/424 (99.5% NS) and 417/417 (100% NS) on *TUB2*, respectively; 536/539 (99.4% NS) and no *RPB2* for *L. euphorbicola*. 

## 3. Discussion

Despite the fact that sampling was restricted to Mexico, this study is the first targeting on *Botryosphaeriaceae* fungi from various palms species and their pathogenic potential. Mexico, as an important hotspot of tropical palm species [38,39], was the unique supplier of palm foliage to German market during the sampling period. The foliage of four out of the five palms species hosted a diversity of five *Botryosphaeriaceae* species. Accurate species identification using multilocus sequence analysis and including *RPB2* region [40] endorsed two novelties among the four delimited *Lasiodiplodia* species. The two novelties are warranted and clearly circumscribed in the current *Lasiodiplodia* phylogeny [37].

The genus *Lasiodiplodia*, similarly to the whole family of *Botryosphaeriaceae*, is characterised by a higher level of cryptic species [37,41,42] and species boundary delimitation of cryptic taxa is difficult to resolve [37]. The first new species described in this study, *L. lodoiceae* isolated from *Lodoicea maldivica*, was resolved as sibling to *L. pontae*. This species was described associated with a necrotic canker on *Anacardium occidentale* and *Spondias purpurea* in Brazil and phylogenetically placed with sister species *L. crassispora* [28]. However, there has been no further record of *L. pontae* until a very recent study showed its strong phylogenetic affinity with the Venezuelan isolate CBS 117454 from *Eucalyptus urophylla* [37]. *L. pontae* also has always formed a monospecific clade [32,37,42]. In our four loci analysis, *L. plurivora* was a sister species to the clade of *L. pontae* and *L. lodoiceae*. Isolates of *L. lodoiceae*, even though clustering to *L. pontae*, formed a clade highly supported by posterior probability (99%) in the analysis of combined four loci. As noted with other phylogenies of *Lasiodiplodia*, Bayesian analysis provided better clade support than maximum parsimony and likelihood [28,37,42]. Low bootstrap support of *Lasiodiplodia* clades has been partly ascribed to missing data [40], as many *Lasiodiplodia* species lack sequences of *TUB2* and mostly *RPB2* loci. 

The second new species, *L. mexicanensis*, isolated from *C. seifrizii* grouped with the Peruvian isolate LACAM1, which was recovered from necrotized branch of *Mango indica* and determined as hybrid of *L. citricola* and *L. parva* [25]. Our combined analysis resolved *L. parva* and *L. mexicanensis* as distinct species forming a clade and *L. citricola* was a sister species to this clade. This result therefore put in question the hybrid character of the isolate LACAM1 that was backed by the shared polymorphism with both *L. citricola* and *L. parva*, solely based on *ITS* and *TEF-1α* [25]. The first previous study suggesting hybridisation in *Lasiodiplodia* proposed four hybrid species, *L. laeliocattleyae*, *L. brasiliense*, *L. missouriana* and *L. viticola* based on the analysis of five genes *ITS*, *TEF-1α*, *TUB2*, *RPB2* and *cmdA* [40]. Nevertheless, if *L. missouriana* has been synonymized to one of its purported parents, *L. gilanensis* [37], the other three hybrid species are further on consistently resolved as distinct species [25,37,42]. Higher genetic similarity characterizing *Lasiodiplodia* species may affect many genes [37,39], thus distinct and distant species as resolved in multilocus analysis, may be essentially different by few nucleotides on a single gene [37]. This seems to be the case for *L. mexicanensis* differing from closely related species *L. parva*, *L. citricola* and *L. euphorbicola* by few nucleotides on *ITS*, *TEF-1α*, *TUB2* and *RPB2* genes. However, the two genes, *ITS* and *TEF-1α* are well-suited for the distinction between *L. mexicanensis* and *L. parva*. Isolate LACAM1 was not available to generate the *RPB2*, but its grouping to *L. mexicanensis* was equally supported in the three loci (*ITS*, *TEF-1α*, *TUB2*) tree (not shown). Isolates from *C. seifrizii* did not form any morph to be used for accurate morphological comparison. It is not common, but some *Lasiodiplodia* isolates may not sporulate under culture conditions [26]. Despite this lack of morphological traits, multiple loci analysis confidently pleaded that these isolates belong together with LACAM1 to a distinct phylogenetic species.

*Botryosphaeriaceae* isolates were mostly (approx. 80%) obtained from necrotic lesions close to blight disease. Except for *L. mexicanensis* recovered from asymptomatic leaves, all other species were isolated from lesions, but from which were also isolated further fungi such as *Colletotrichum*, *Pestalotiopsis*, *Fusarium*, *Alternaria* usually causing blight disease [10,11,12,43]. Determining fungi causing these blight symptoms was not part of the focus of this study. However, at least *L. brasiliensis*, isolated in this study, is implicated in leaf blight disease in palms. It causes, together with other *Lasiodiplodia* species, the blight disease of *Cocos nucifera* [44,45] and is also involved in the postharvest stem-end rot, considerably damaging fruits of this host [17,44,45]. Furthermore, *L. brasiliensis* first described associated with papaya stem-end rot in Brazil [22] is now plurivorous and geographically widespread, causing cankers, dieback and blights on diverse woody and crop plants [18,23,25,46,47,48]. *L. euphorbicola* hitherto not reported on palm is equally plurivorous, widespread in Brazil causing dieback, collar and root rot, gummosis, cladode brown spot on crop plants [20,21,23,24,49]. Its geographical distribution includes West Africa and further countries such as Botswana, Madagascar, Namibia and Zimbabwe where it affects *Adansonia digitata* [39]. Surprisingly, despite the host diversity examined, the most common *L. theobromae* colonising plants, including many *Arecaceae*, in the warm regions [19,50] was not recovered. Instead, the predominantly endophytic *E. endophytica* was the most frequent species. It has shown weak aggressiveness on young *Terminalia mantaly* and *T. catappa* [51].

In light of this knowledge on the recorded *Botryosphaeriaceae* species as plant pathogens, trials were conducted and suggested a potential risk on apple and pear, two important temperate pome fruit species. All six *Botryosphaeriaceae* fungi were able to cause rot damage on both fruits under the experimental conditions. Hence, it can be expected that, if all these species are able to successfully establish in Europe, they may be regarded as a potential threat at least inducing fruit rot disease on apple and pear. The geographical range of *L. theobromae*, the northernmost distributed fruit-rotting *Lasiodiplodia* already includes Europe [52,53,54]. It is the most common *Lasiodiplodia* species associated with pre- and postharvest fruit diseases, with the most common being the stem-end rot [55,56]. *L. theobromae* belongs to the *Botryosphaeriaceae* fungi complex causing the severe fruit rot of grapevines with great adaptation to Northern Hemisphere [54]. Therefore, there is a likelihood that *Botryosphaeriaceae* fungi associated with palm foliage can also establish and spread in Europe. Furthermore, increasing temperature as an immediate consequence of the ongoing climate change may be a critical factor in determining the successful settling [57,58,59].

This study unveiled the potential of tropical palm foliage as source of crop plant pathogenic *Botryosphaeriaceae* fungi and as pathways facilitating their movement. These *Botryosphaeriaceae* fungi represent a tiny part of the fungal community in palm foliage. Combined culture method and analysis of metagenomic data also recovered fungi of the families *Pestalotiopsidaceae*, *Glomerellaceae*, *Nectriaceae* comprising notorious plant pathogens with many species exotic to Europe (data not published). It is to be noted that the movement of plant material, even in compliance with strict requirements, can always create undesirable effects. Indeed, it is not possible within the scope of border control to carry out inclusive investigation for all harmful organisms (fungi, bacteria, viruses and insects). Further undertakings on the overall biological community of palm foliage are required and will enable researchers to comprehensively size up the phytosanitary risks.

## 4. Materials and Methods

### 4.1. Sampling and Fungal Isolation

Palm foliage sampling was conducted between June 2017 and February 2018 and consisted of taking thee bunches of foliage from each of the 12 consignments, for a total of 36 foliage bunches, all originating from Mexico. Samples often presented diverse disease symptoms and included five palm species: *Chamaedorea elegans*, *C. metallica*, *C. seifrizii*, *Dypsis lutescens* and *Lodoicea maldivica*. Sampled tissues were surface sterilized in 70% ethanol for 1 min, in 1.5% sodium hypochlorite for 5 min, in 70% ethanol for 30 s and finally rinsed twice (5 min each) in sterile distilled water. In total 648 pieces of approximately 5 mm were cut from both asymptomatic and symptomatic (when present) tissues and placed (5 pieces) on each MEA plate amended with 1% tetracycline. Plates were incubated at 25 °C with a 12 h photoperiod for 4 days. Growing colonies were transferred onto new MEA plate. Isolates with cottony and grey, greyish-green to black tones resembling those of *Botryosphaeriaceae* fungi were subcultured on 2% water agar (WA) or SNA medium amended with sterilized pine needles and incubated (4–5 weeks) at 25 °C under near-ultraviolet light with 12 h photoperiod. From sporulating colonies, single-spore cultures were obtained on potato dextrose agar (PDA) according to [60]. Nonsporulating isolates underwent *ITS* sequencing for confirming the assignment to *Botryosphaeriaceae*. Representative isolates of all species were deposited in the culture collection of the Federal Research Centre for Cultivated Plants (JKI), Braunschweig, Germany, and the ex-type living cultures of the new species were additionally deposited at the Leibnitz Institute DSMZ-German Collection of Microorganisms and Cell Cultures, Braunschweig.

### 4.2. Morphocultural Characterization of Isolates 

Isolates were further grouped into morphotypes based on growth rate and characters of conidia. Representative isolates of morphotypes were used for morphocultural characterization carried out on 9-cm-diameter PDA plates and incubated for 10 d at 25 °C and 35 °C with a 12 h photoperiod. Colony radius was measured daily from the 3rd to the 10th day in 2 perpendicular directions and was used to calculate the mycelial growth rate (mm d^−1^). Three replicates for each isolate were used and the assay was repeated twice. Conidiomata were formed between 2–4 wk. Photographs of cultures were made with a Canon EOS 60D. Microscopic characters were observed and measured in lactic acid using differential interference contrast (DIC) illumination on a Leica DM 5500 B microscope, and the images were captured using a Leica DFC 550 digital camera (400×) coupled to the microscope. Average measure of each microscopic structure was calculated from 50 measurements.

### 4.3. Phylogenetic Characterization of Isolates

#### 4.3.1. DNA Extraction, PCR Amplification, and Sequencing 

Mycelium was taken from 5-day-old colonies grown on PDA at 25 °C and used for extraction of the genomic DNA with Quiagen Plant Mini Kit (QUIAGEN) following the manufacturer’s protocol. Four loci, *ITS rDNA*, part of the translation elongation factor 1α (*TEF-1α*), partial b-tubulin (*TUB2*) and RNA polymerase subunit II (*RPB2*) gene regions, were amplified by PCR reactions, with the primer pairs ITS1f [61] and ITS4 [62], EF1-728F and EF1-986R [63], Bt-2a and Bt-2b [64] and rpb2-LasF and rpb2-LasR [39], respectively. PCR reactions were performed using cycling conditions as indicated in [39]. PCR products were checked on 1% agarose electrophoresis gel stained with ROTI^®^GelStain (Roth, Germany). Amplicons were purified with QIAquick PCR Purification Kit (QUIAGEN) and sequenced with the PCR primers in both directions by Macrogen Europe (Netherlands). Raw nucleotide sequences were edited in MEGA v. 7 [65].

#### 4.3.2. Phylogenetic Analyses 

The *ITS* and *TEF-1α* genes of representative isolates of morphotypes were initially sequenced. *TEF-1α* sequences were assembled into contigs with 98% similarity using Sequencher 5.0. and representative OTUs used for BLAST searches in NCBI GenBank (accessed 15.04.2021). *TEF-1α* dataset including sequences of ex-type isolates were analysed to reveal primary placement of isolates within *Botryosphaeriaceae* genera. Based on these results, *TUB2* and *RPB2* sequences were then generated for morphotype representatives for multilocus analysis. Genes were concatenated for constructing two multigene phylogenies using *ITS* and *TEF-1α* genes for *Endomelanconiopsis* and *ITS*, *TEF-1α*, *TUB2*, *RPB2* genes for *Lasiodiplodia*. Sequences were aligned in MAFFT v. 7 online version [66] and manually optimized with MEGA v. 7. Phylogenetic trees were constructed using the maximum parsimony (MP), maximum likelihood (ML) and Bayesian inference (BI) methods. MP analysis conducted in PAUP v. 4.0b10 [67] used heuristic searches with Tree Bisection-Reconnection (TBR), MAXTREES set to autoincrease, saving 10 trees per replicate and clade stability assessed with 1000 bootstrap replicates. ML analyses were performed using RAxML-HPC Blackbox version 8.2.10 [68] as implemented on the CIPRES Science Gateway [69] with estimate proportion of invariable sites GTRGAMMA+I and branch support evaluated by 1000 bootstrap replicates. Bayesian inference was carried out in MrBayes v. 3.2 [70]. The best substitution model for tree reconstruction was estimated by both the Akaike information criterion and the Bayesian information criterion in jModelTest 2.0 [71]. Four Markov Chain Monte Carlo chains were run simultaneously starting from random trees for 5,000,000 generations and sampling every 100th generation. After discarding the first 25% of trees as burn-in phase, a consensus Bayesian tree and Bayesian posterior probabilities (BPP) were determined based on all remaining trees. A BPP above 0.90 was considered as significant value. The individual phylogenies were constructed using ML analyses as described above. Number of sequences aligned varied among genes due to many missing data in *TUB2* and mostly *RPB2*. Trees were visualized and further edited in TreeGraph 2 [72]. New sequences generated in this study were deposited in GenBank (www.ncbi.nlm.nih.gov) and accession numbers are shown in Table S1 together with those retrieved from GenBank. Resulting alignments and trees have been deposited in TreeBASE (https://www.treebase.org/treebase, Submission: 28655).

### 4.4. Pathogenicity Test

*Botryosphaeriaceae* species are associated worldwide with postharvest disease and mostly fruit stem-end rot. Therefore, pathogenicity trials were conducted on fruits of *Malus domestica* (apple) and *Pyrus communis* (European pear). Both are two important pome fruits already affected by different rots and postharvest disorders during storage [73]. We selected pear variety “Comtesse de Paris” and apple variety “Goldparmäne” and fruits without visible signs of disease and fungicide treatment. Two isolates of each identified *Botryosphaeriaceae* fungi were used to inoculate the fruits. Fruits were first washed and surface sterilized with 1.2% sodium hypochlorite for 5 min, rinsed twice with sterile distilled water and left dry on paper filter prior to inoculation. Fruits were wounded to a 3-mm depth at two sites towards both ends. On the upper wound was placed a 3-mm diameter PDA agar plug containing 5-days-old mycelium of the isolate to be tested. Similarly, on the below wound was placed a sterile non-colonised 3-mm PDA agar plug as control. Inoculated fruits were placed each on one 90 × 14.5 mm petridish round and arranged in plastic trays equipped with lid and the bottom covered with layers of moistened sterile kitchen roll. Incubation was carried out in a thermostatically controlled incubator at 25 °C with a 12 h photoperiod. Plastic trays were first kept closed for 2 days and subsequently open for further 6 days.

After the third day of incubation, length of lesion appearing on each fruit was measured daily in two perpendicular directions until the eighth day and data used for calculating the lesion size (mm) and growth rate (mm day^−1^). The experiment was conducted in randomized design with two fruits per replicate, and there were three replicates per isolate. At the end of each replicate, Koch’s postulate was verified by fungal re-isolation from necrotic tissue from each lesion. Mean values of lesions were analysed using One-way analysis of variance (ANOVA) followed by *t*-Test at a = 0.05 in Microsoft Excel 2010.

## Data Availability

All sequence data are available in NCBI GenBank (https://www.ncbi.nlm.nih.gov/) following the accession numbers in the Supplementary Materials (Table S1). Datasets and phylogenetic trees are available in TreeBase (Submission: 28655).

## Supplementary Materials

The following are available online at https://www.mdpi.com/article/10.3390/pathogens10101297/s1, Figures S1–S4: RAxML tree of *Lasiodiplodia* based on *ITS* (Figure S1), *TEF-1α* (Figure S2), *TUB2* (Figure S3), *RPB2* (Figure S4), datasets showing the phylogenetic placement of *Lasiodiplodia* isolates from palm foliage from Mexico. Maximum likelihood bootstrap values (MLB > 60%) are displayed at the nodes. The trees were rooted to *Diplodia mutila* (CMW7060) and *Diplodia seriata* (CBS 112555). Isolates from palm foliage are in bold. Table S1: Culture accession numbers, location, host and GenBank accession numbers of *Botryosphaeriaceae* isolates included in this study.
