# Supplementary material for: Palm Foliage as Pathways of Pathogenic Botryosphaeriaceae Fungi and Host of New Lasiodiplodia Species from Mexico
Source: Pathogens. 2021 Oct 8;10(10):1297. doi: 10.3390/pathogens10101297 (PMC8541634; doi:10.3390/pathogens10101297)
Supplement: Supplementary file 1 [file pathogens-10-01297-s001.zip › pathogens-1360540-supplementary-final.pdf]

Palm Foliage as Pathways Of Pathogenic Botryosphaeriaceae Fungi And Host of New *Lasiodiplodia* Species from Mexico

Clovis Douanla-Meli[1*], Andreas Scharnhorst[2]

[1] Julius Kühn Institute (JKI) – Federal Research Centre for Cultivated Plants, Institute for National and International Plant Health, Messeweg 11-12, 38104 Braunschweig, Germany (https://*orcid*.org/*0000-0001-7182-5905)*; clovis.douanla-meli@julius-kuehn.de

[2] Dezernat 51.4, Regierungspräsidium Gießen Pflanzenschutzdienst Hessen, Flughafen Frankfurt; e-mail@e-mail.com

* Correspondence: clovis.douanla-meli@julius-kuehn.de; Tel.: +49 5312994370, CDM)

Supplementary files

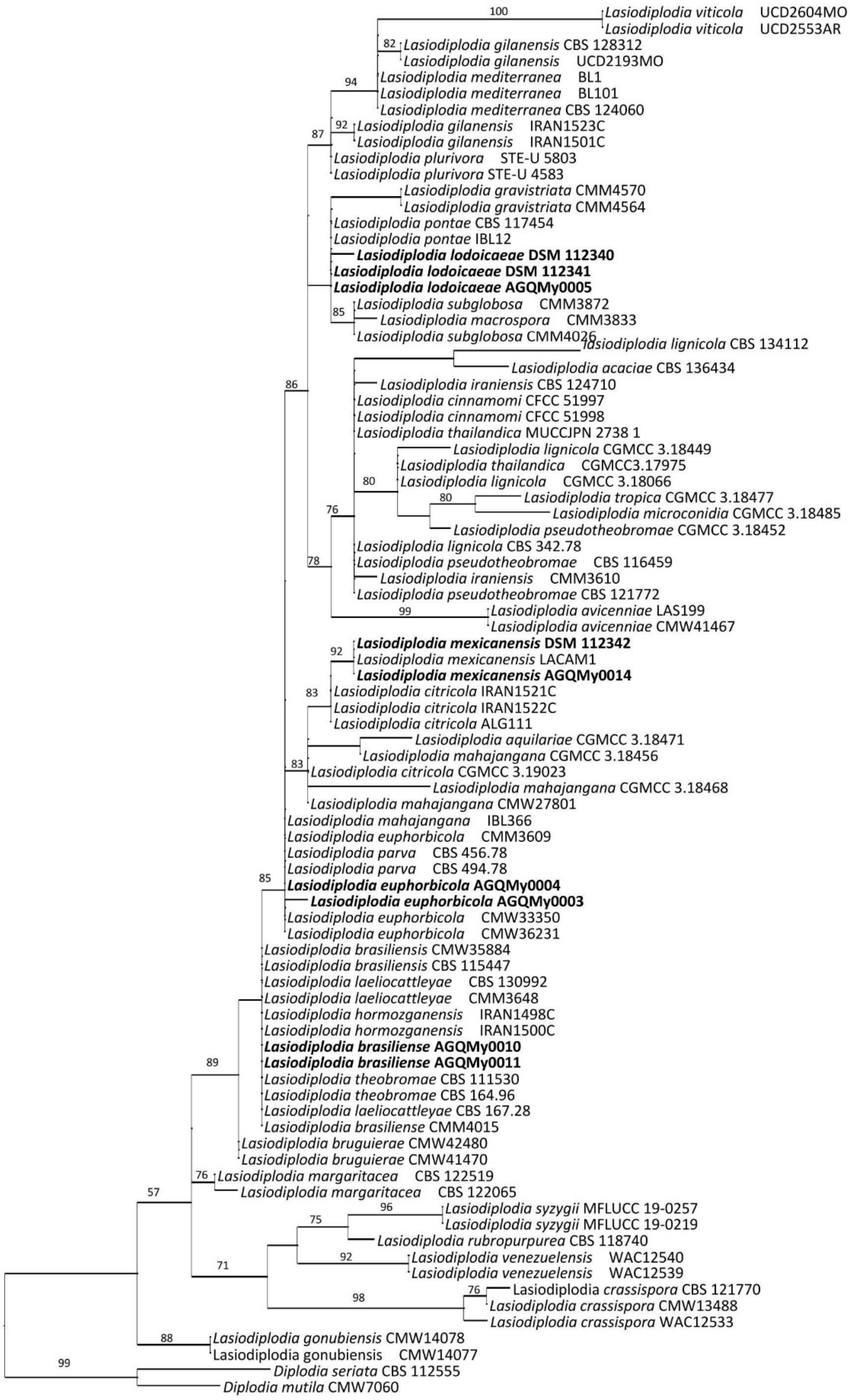

Figure S1. RAxML tree of *Lasiodiplodia* based on *ITS* dataset showing the phylogenetic placement of isolates from palm foliage from Mexico. Maximum likelihood bootstrap values (MLB > 60 %) displayed at the nodes. The tree is rooted to *Diplodia mutila* (CMW7060) and *Diplodia seriata* (CBS 112555). Isolates from palm foliage are in bold characters.

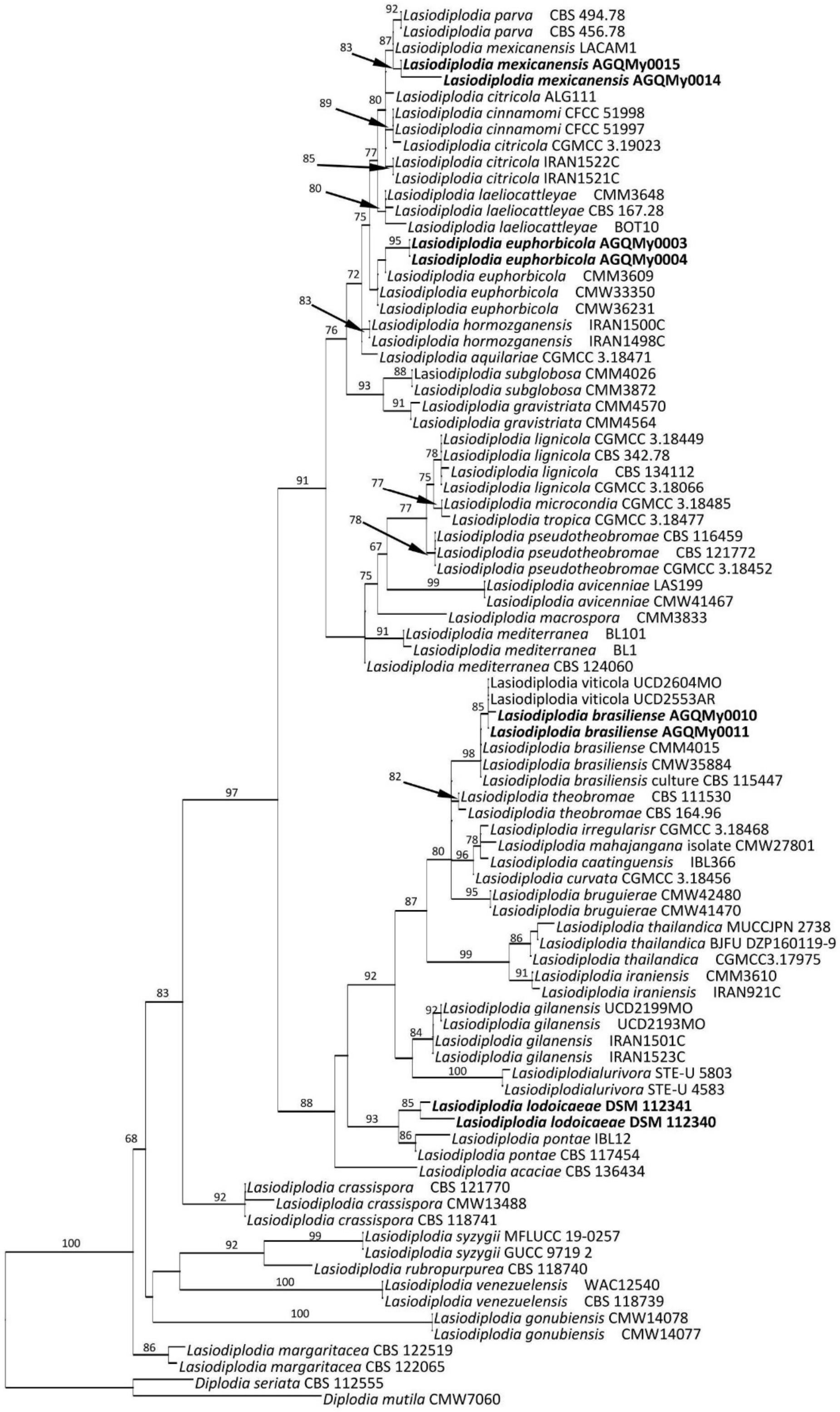

Figure S2. RAxML tree of *Lasiodiplodia* based on *TEF-1α* dataset showing the phylogenetic placement of isolates from palm foliage from Mexico. Maximum likelihood bootstrap values (MLB > 60 %) displayed at the nodes. The tree is rooted to *Diplodia mutila* (CMW7060) and *Diplodia seriata* (CBS 112555). Isolates from palm foliage are in bold characters.

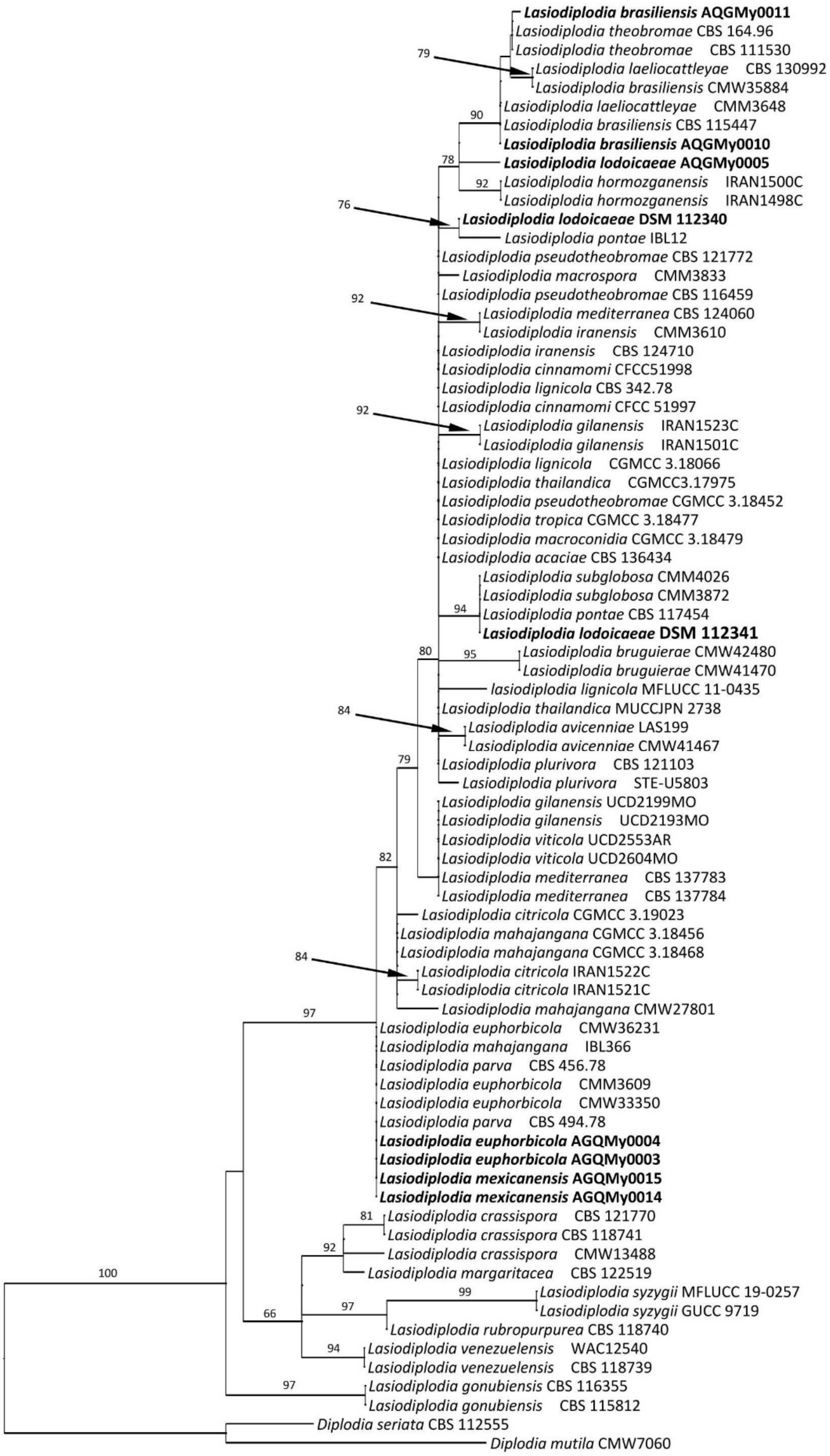

Figure S3. RAxML tree of *Lasiodiplodia* based on *TUB2* dataset showing the phylogenetic placement of isolates from palm foliage from Mexico. Maximum likelihood bootstrap values (MLB > 60 %) displayed at the nodes. The tree is rooted to *Diplodia mutila* (CMW7060) and *Diplodia seriata* (CBS 112555). Isolates from palm foliage are in bold characters.

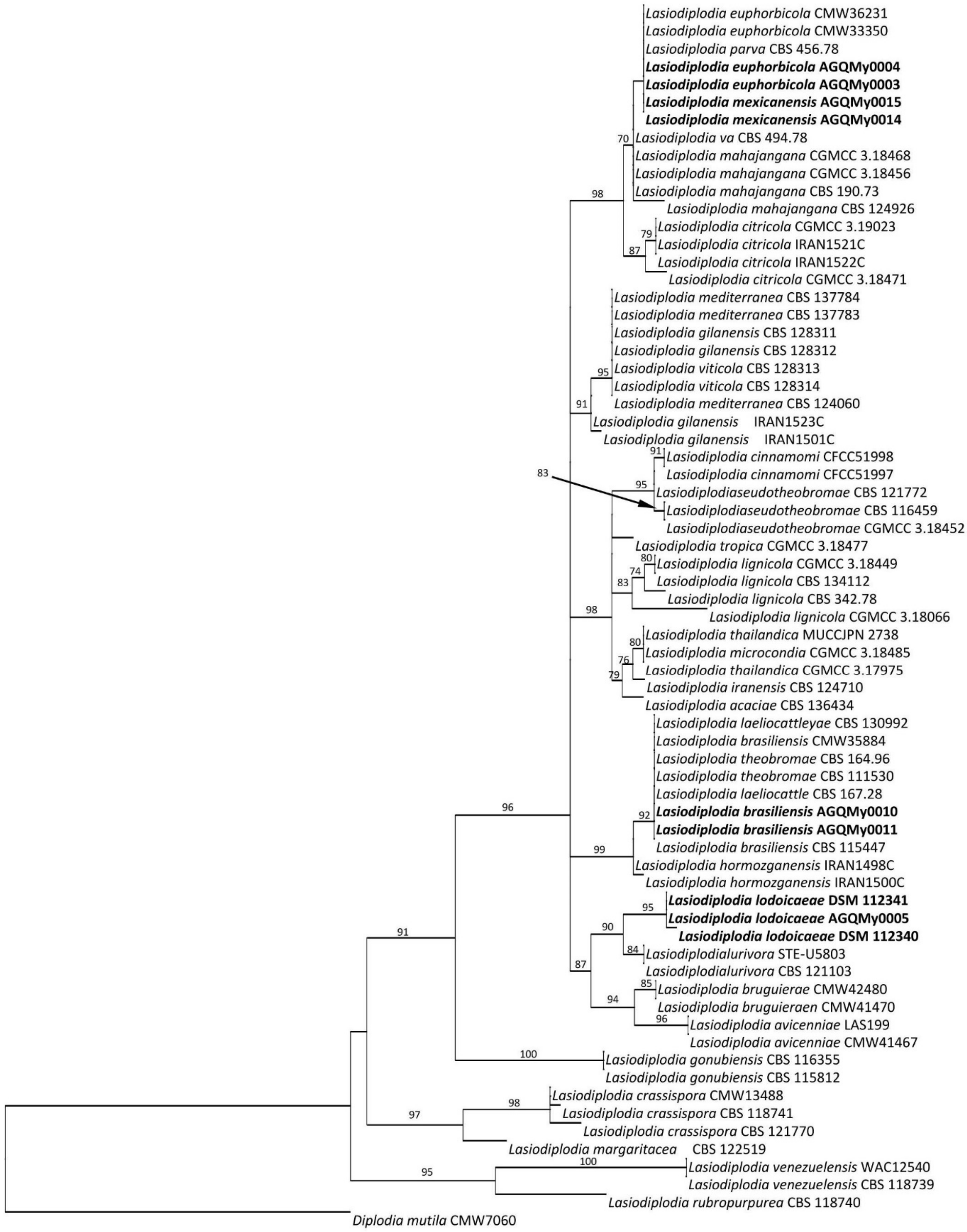

Figure S4. RAxML tree of *Lasiodiplodia* based on *RPB2* dataset showing the phylogenetic placement of isolates from palm foliage from Mexico. Maximum likelihood bootstrap values (MLB > 60 %) displayed at the nodes. The tree is rooted to *Diplodia mutila* (CMW7060) and *Diplodia seriata* (CBS 112555). Isolates from palm foliage are in bold characters.

Table S1. Culture accession numbers, location, host and GenBank accession numbers of Botryosphaeriaceae isolates included in this study. The newly generated sequences are in bold.

Species	Isolate code	Location	Host	Genbank accession number			
				ITS	TEF-1α	TUB2	RPB2
Diplodia mutila [T]	CMW 7060	Netherlands	*Fraxinus excelsior*	AY236955	AY236904	AY236933	EU339574
D. seriata [T]	CBS 112555	Portugal	*Vitis vinifera*	AY259094	AY573220	DQ458856	N/A
Endomelanconiopsis endophytica [T]	CBS 120397	Panama	*Theobroma cacao*	NR_156272	KF766164	N/A	N/A
E. endophytica	**AGQMy0007**	**Mexico**	***Lodoicea maldivica***	**MW274143**	**MW604226**	N/A	N/A
E. endophytica	**AGQMy0008**	**Mexico**	***Lo. maldivica***	**MW274149**	**MW604232**	N/A	N/A
E. endophytica	**AGQMy0009**	**Mexico**	***Dypsis lutescens***	**MW274142**	**MW604231**	N/A	N/A
E. endophytica	**AGQMy0012**	**Mexico**	***D. lutescens***	**MW274141**	**MW604225**	N/A	N/A
E. endophytica	CBS 122542	Panama	*Heisteria concinna*	EU683669	EU683650	N/A	N/A
E. endophytica	CBS 122547	Panama	*T. cacao*	EU683666	EU683647	N/A	N/A
E. endophytica	CBS 122552	Panama	*H. concinna*	EU683667	EU683648	N/A	N/A
E. endophytica	CBS 122551	Panama	*T. cacao*	EU683665	EU683646	N/A	N/A
E. endophytica	9188	Panama	*H. concinna*	EU683668	EU683649	N/A	N/A
E. endophytica	CBS 122550	Panama	*T. cacao*	EU683664	EU683645	N/A	N/A
E. endophytica	CMW28551	Cameroon	*Terminalia superba*	GQ469967	GQ469907	N/A	N/A
E. endophytica	CMW28552	Cameroon	*Te. superba*	GQ469968	GQ469908	N/A	N/A
E. endophytica	CMW28618	Cameroon	*Te. ivorensis*	GQ469966	GQ469906	N/A	N/A
E. microspora	CBS 353.97	Papua New Guinea	Soil	EU683655	EU683636	N/A	N/A
E. microspora	580809	China	plant root	MK371761	N/A	N/A	N/A
E. microspora	PP108	Peru	*Hevea brasiliensis*	FJ884091	N/A	N/A	N/A
E. microspora	PP73 18S	Peru	*H. brasiliensis*	FJ884092	N/A	N/A	N/A
E. microspora [T]	CBS 353.97	Papua New Guinea	Soil	EU683655	EU683636	N/A	N/A
E. freycinetiae [T]	MFLUCC 17-0547	Thailand	Pandanaceae	MG646955	MG646983	N/A	N/A
E. freycinetiae	BSNB-0528	French Guiana	*Astrocaryum sciophilum*	MK650407	N/A	N/A	N/A
E. freycinetiae	BSNB-0587	French Guiana	*A. sciophilum*	MK650386	N/A	N/A	N/A
E. freycinetiae	BSNB-0681	French Guiana	*A. sciophilum*	MK650386	N/A	N/A	N/A
E. freycinetiae	BSNB-0547	French Guiana	*A. sciophilum*	MK650363	N/A	N/A	N/A
Lasiodiplodia sp.	LACAM1	Peru	*Mangifera indica*	KU507469	KU507436	N/A	N/A
Lasiodiplodia acaciae	CBS 136434	Indonesia	*Acacia* sp., leaf spot	MT587421	MT592133	MT592613	MT592307
L. avicenniae [T]	CBS 139670/CMW41467	South Africa	*Avicennia marina*	KP860835	KP860680	KP860758	KU587878
L. avicenniae	LAS199	South Africa	*A. marina*	KU587957	KU587947	KU587868	KU587880
L. brasiliense [T]	CMM 4015	Brazil	*M. indica*	JX464063	JX464049	N/A	N/A
L. brasiliense	CMW 35884	Laos	*A. crassna*	KU887094	KU886972	KU887466	KU696345
L. brasiliense	CBS 115447	Hong Kong	*Psychotria tutcheri*	MT587422	MT592134	MT592614	MT592308
L. brasiliense	**AGQMy0010**	**Mexico**	***Lo. maldivica***	**MW274144**	**MW604227**	**MW604237**	**MW604216**
L. brasiliense	**AGQMy0011**	**Mexico**	***Lo. maldivica***	**MW274145**	**MW604228**	**MW604238**	**MW604217**
L. bruguierae [T]	CBS 139669/ CMW41470	South Africa	*Bruguiera gymnorrhiza*	KP860834	KP860679	KP860757	KU587875
L. bruguierae	CMW42480	South Africa	*B. gymnorrhiza*	KP860832	KP860677	KP860755	KU587876
L. cinnamomi [T]	CFCC 51997	China	*Cinnamomum camphor*	MG866028	MH236799	MH236797	N/A
L. cinnamomi	CFCC 51998	China	*C. camphora*	MG866029	MH236800	MH236798	N/A
L. citricola [T]	CBS 124707/ IRAN1522C	Iran	*Citrus* sp.	GU945354	GU945340	KU887505	KU696351
L. citricola	IRAN1521C	Iran	*Citrus* sp.	GU945353	GU945339	KU887504	KU696350

L. crassispora ^{T}	CBS 118741/WAC12533	Australia	*Santalum album*	DQ103550	EU673303	KU887506	KU696353
L. crassispora	CMW 13488	Venezuela	*Eucalyptus urophylla*	DQ103552	DQ103559	KU887507	KU696352
L. crassispora	CBS 121770	Namibia	*Acacia mellifera*	EU101307	EU101352	KU887527	KU696378
L. euphorbiicola ^{T}	CMM 3609	Brazil	*J. cuscas*	KF234543	KF226689	KF254926	N/A
L. euphorbiicola	CMW 33350	Botswana	*Adansonia digitata*	KU887149	KU887026	KU887455	KU696346
L. euphorbiicola	CMW 36231	Zimbabwe	*A. digitata*	KU887187	KU887063	KU887494	KU696347
L. euphorbiicola	**AGQMy0003**	**Mexico**	***Chamaedorea elegans***	**MW274139**	**MW604223**	**MW604235**	**MW604214**
L. euphorbiicola	**AGQMy0004**	**Mexico**	***Ch. elegans***	**MW274140**	**MW604224**	**MW604236**	**MW604215**
L. gilanensis ^{T}	CBS 124704 /IRAN1523C	Iran	Unknown	GU945351	GU945342	KU887511	KU696357
L. gilanensis	CBS 124705	Iran	Unknown	GU945352	GU945341	KU887510	KU696356
L. gonubiensis ^{T}	CBS 115812 /CMW14077	South Africa	*Syzygium cordatum*	AY639595	DQ103566	DQ458860	KU696359
L. gravistriata ^{T}	CMM 4564	Brazil	*Anacardium humile*	KT250949	KT250950	N/A	N/A
L. gravistriata	CMM 4570	Brazil	*A. humile*	KT250948	KT266814	N/A	N/A
L. hormozganensis ^{T}	IRAN1500C	Iran	*Ole* asp.	GU945355	GU945343	KU887515	KU696361
L. hormozganensis	IRAN 1498C	Iran	*M. indica*	GU945356	GU945344	KU887514	KU696360
L. iraniensis ^{T}	CBS 124710/ IRAN921	Iran	*M. indica*	GU945346	GU945334	KU887516	KU696363
L. iraniensis	CMM 3610	Brazil	*J. curcas*	KF234544	KF226690	KF254927	N/A
L. laeliocattleyae ^{T}	CBS 130992/BOT10	Egypt	*M. indica*	JN814397	JN814424	KU887508	KU696354
L. laeliocattleyae	CMM3648	Brazil	*Jatropha curcas*	KF234549	KF226705	KF254933	N/A
L. laeliocattleyae	CBS 167.28	Italy	*Laelio cattleya*	MT587425	MT592136	MT592618	MT592313
L. lignicola ^{T}	CBS 134112	Thailand	dead wood	JX646797	KU887003	JX646845	KU696364
L. lignicola	CGMCC 3.18449	Laos	*A. crassna*	KY783466	KY848619	N/A	KY848586
L. lignicola	CBS 342.78	Germany	*Sterculia oblonga*	KX464140	KX464634	KX464908	KX463989
L. loidaceae ^{T}	**DSM 112340**	**Mexico**	***Lo. maldivica***	**MW274148**	**MW604230**	**MW604240**	**MW604219**
L. loidaceae	**AGQMy0005**	**Mexico**	***Lo. maldivica***	**MW274147**	**N/A**	**MW604241**	**MW604220**
L. loidaceae	**DSM 112341**	**Mexico**	***Lo. maldivica***	**MW274146**	**MW604229**	**MW604239**	**MW604218**
L. macroconidia ^{T}	CGMCC 3.18479	Laos	*A. crassna*	KY783438	KY848597	KY848530	KY848558
L. macrospora ^{T}	CMM 3833	Brazil	*J. curcas*	KF234557	KF226718	KF254941	N/A
L. mahajangana ^{T}	CBS 124927/CMW27818	Madagascar	*Te. catappa*	FJ900595	FJ900641	FJ900630	KU696366
L. mahajangana	CBS 190.73	Tanzania	*Persea americana*	EF622068	EF622048	MT592616	MT592311
L. mahajangana	*CGMCC 3.18456*	Laos	*A. crassna*	KY783437	KY848596	KY848529	KY848557
L. mahajangana	CGMCC 3.18477	Laos	*A. crassna*	KY783454	KY848616	KY848540	KY848574
L. margaritacea ^{T}	CBS 122519	Australia	*Adansonia gibbosa*	EU144050	EU144065	KU887520	KU696367
L. margaritacea	CBS 122065	Australia	*A. gibbosa*	EU144051	EU144066	N/A	N/A
L. mediterranea ^{T}	CBS 137783 / BL1	Italy	*Quercus ilex*	KJ638312	KJ638331	KU887521	KU696368
L. mediterranea	CBS 137784	Italy	*Quercus ilex*	KJ638311	KJ638330	KU887522	KU696369
L. mediterranea	CBS 124060	Italy	*V. vinifera*	KX464148	KX464642	KX464917	KX463994
L. mexicanense ^{T}	**DSM 112342**	**Mexico**	***Ch. seifrizii***	**MW274151**	**MW604234**	**MW604243**	**MW604222**
L. mexicanense	**AGQMy0015**	**Mexico**	***Ch. seifrizii***	**MW274150**	**MW604233**	**MW604242**	**MW604221**

L. mexicanense	LACAM1	Peru	*M. indica*	KU507469	KU507436	N/A	N/A
L. microcondia T	CGMCC 3.18485	China	*A. crassna*	KY783441	KY848614	N/A	KY848561
L. parva T	CBS 456.78	Colombia	cassava field soil	EF622083	EF622063	KU887523	KU696372
L. parva	CBS 494.78	Colombia	cassava field soil	EF622084	EF622064	EU673114	KU696373
L. plurivora T	STE-U 5803	South Africa	*Prunus salicina*	EF445362	EF445395	KP872421	KP872479
L. plurivora	STE-U 4583	South Africa	*Vitis vinifera*	AY343482	EF445396	KU887525	KU696375
L. pontae T	CMM 1277	Brazil	*S. purpurea*	KT151794	KT151791	KT151797	N/A
L. pontae	CBS 117454	Venezuela	*E. urophylla*	MT587432	MT592144	MT592626	N/A
L. pseudotheobromae T	CBS 116459	Costa Rica	*Gmelina arborea*	EF622077	EF622057	EU673111	KU696376
L. pseudotheobromae	CGMCC 3.18452	China	*A. crassna*	KY783467	KY848620	KY848549	KY848587
L. pseudotheobromae	CBS 121772	Namibia	*Acacia mellifera*	EU101310	EU101355	**MT592627**	**MT592323**
L. rubropurpurea T	CBS 118740	Australia	*Eucalyptus grandis*	DQ103553	EU673304	EU673136	KU696380
L. rubropurpurea	WAC 12536 = CMW 15207	Australia	*Eucalyptus grandis*	DQ103554	DQ103572	KU887530	KU696381
L. subglobosa T	CMM 3872	Brazil	*J. curcas*	KF234558	KF226721	KF254942	N/A
L. subglobosa	CMM 4046	Brazil	*J. curcas*	KF234560	KF226723	KF254944	N/A
L. syzygii T	MFLUCC 19-0219.1	Thailand	*Sy. samarangense*	MT990531	MW016943	MW014331	N/A
L. syzygii	GUCC 9719.2	Thailand	*Sy. samarangense*	MW081991	MW087101	MW087104	N/A
L. thailandica T	CGMCC 3.18384	China	*Albizia chinensis*	KY767663	KY751304	KY751301	N/A
L. thailandica	CGMCC 3.17975	China	*Acacia confusa*	KX499879	KX499917	KX499992	KX499955
L. thailandica	BJFU DZP160119-9	China	*Podocarpus macrophyllus*	KY676788	KY751303	KY676794	N/A
L. theobromae T	CBS 164.96	New Guinea	Fruit on coral reef coast	AY640255	AY640258	KU887532	KU696383
L. theobromae	CBS 111530	Unknown	Unknown	EF622074	EF622054	KU887531	KU696382
L. venezuelensis T	WAC12539	Venezuela	*Acacia mangium*	DQ103547	EU673305	KU887533	KP872490
L. venezuelensis	WAC 12540	Venezuela	*A. mangium*	DQ103548	DQ103569	KU887534	KP872491
L. viticola T	UCD2604MO	USA	*V. vinifera*	HQ288228	HQ288270	HQ288307	KU696386
L. viticola	UCD2553AR	USA	*V. vinifera*	HQ288227	HQ288269	HQ288306	KU696385
Phyllosticta parthenocissi T	CBS 111645T	USA	*Parthenocissus quinquefolia*	NR_147322	JN692530	N/A	N/A

T Ex-type strains, N/A: not available.
